# Dried blood spots: Effects of less than optimal collection, shipping time, heat, and humidity

**DOI:** 10.1002/ajhb.23390

**Published:** 2020-01-10

**Authors:** Eileen M. Crimmins, Yuan S. Zhang, Jung Ki Kim, Stephen Frochen, Hyewon Kang, Hyunju Shim, Jennifer Ailshire, Alan Potter, Jake Cofferen, Jessica Faul

**Affiliations:** ^1^ Andrus Gerontology Center University of Southern California Los Angeles California; ^2^ Carolina Population Center University of North Carolina Chapel Hill North Carolina; ^3^ Department of Laboratory Medicine University of Washington Seattle Washington; ^4^ Survey Research Center University of Michigan Ann Arbor Michigan

## Abstract

**Objectives:**

This study investigates how factors related to collection, storage, transport time, and environmental conditions affect the quality and accuracy of analyses of dried blood spot (DBS) samples.

**Methods:**

Data come from the 2016 Health and Retirement Study (HRS) DBS laboratory reports and the HRS merged with the National Climatic Data Center (NCDC) Global Historical Climate Network Daily (NCDC GHCN‐Daily) and the NCDC Local Climatological Data, by zip code. We ran regression models to examine the associations between assay values based on DBS for five analytes (total cholesterol, high‐density lipoprotein (HDL) cholesterol, glycosylated hemoglobin (HbA1c), C‐reactive protein (CRP), and cystatin C) and the characteristics of DBS cards and drops, shipping time, and temperature, and humidity at the time of collection.

**Results:**

We found cholesterol measures to be sensitive to many factors including small spots, shipping time, high temperature and humidity. Small spots in DBS cards are related to lower values across all analytes. Longer DBS transit time before freezing is associated with lower values of total and HDL cholesterol and cystatin C. Results were similar whether or not venous blood sample values were included in equations.

**Conclusions:**

Small spots, long shipping time, and exposure to high temperature and humidity need to be avoided if possible. Quality of spots and cards and information on shipping time and conditions should be coded with the data to make adjustments in values when necessary. The different results across analytes indicate that results cannot be generalized to all DBS assays.

## 1 INTRODUCTION

Dried blood spots (DBSs) have been used increasingly in recent years as an alternative to traditional venous blood collection in many large population studies (McDade, Burhop, & Dohnal, 2004; McDade, Williams, & Snodgrass, 2007; Weir, 2008). This minimally invasive blood collection method is relatively simple compared to conventional blood sampling as it can be done by interviewers rather than medically trained personnel (Amsterdam & Waldrop, 2010; Deep, Kumar, Kumar, & Thakkar, 2012). DBS samples may also be easier to obtain as respondents are more likely to agree to participate (Samuelsson et al., 2015; Williams & McDade, 2009). Additional desirable features of this type of blood collection include minimal requirements for transport and storage (e.g., ambient temperature, no dry ice required, no hazardous material regulations in shipping), and reduced biohazard risks and contamination (Kulmatycki, Xu, & Jarugula, 2014; Mei & Lee, 2014; Parker & Cubitt, 1999; Zhang, Majumdar, Flarakos, & Tse, 2013). In general, blood collection using this approach is simpler and less costly. It is also thought that analytes in DBS samples can be preserved for long periods without deterioration and DBS involves lower cost than venous blood samples (VBSs) (Hannon & Therrell, 2014).

While there are many advantages in DBS sampling, conditions during the collection, shipping, handling, and preanalytic phase such as the quality of the spots and cards, time without freezing and atmospheric conditions may have substantial influences on the validity of DBS assays. For instance, smaller blood spots have been found to yield lower results for all analytes, raising risks of significant negative biases with the use of DBS; and the effect of multispotted samples rather than single spots differed depending on the analyte (George & Moat, 2016). Length of time before freezing may also affect the quality of DBS assays. Enzyme activities in DBS have been found to show considerable differences depending on storage method, the use of desiccant, and the duration of shipping (Elbin et al., 2011). Storage temperature is another factor that affects the stability of the DBS samples; DBS samples stored at 20°C showed significantly greater deterioration over a year compared to samples stored at 4°C or below 0°C for the same duration (Batterman & Chernyak, 2014). Multiple freeze‐thaw cycles have also been documented to cause significant variations in lipid concentrations compared to fresh samples (Zivkovic et al., 2009).

Environmental conditions at the time of collection could also affect the values of DBS assays as DBS are susceptible to high temperature and humidity (Zhang et al., 2013), which can facilitate bacterial growth or enhance the rate of analyte degradation (Quraishi, Jain, & Ambekar, 2013; Sharma, Jaiswal, Shukla, & Lal, 2014). High temperature has been documented to be particularly detrimental for high‐density lipoprotein (HDL) cholesterol (Halonen, Zanobetti, Sparrow, Vokonas, & Schwartz, 2011). High humidity and temperature appear to have an interactive effect on analytes (Freer, 2005; García‐Lerma et al., 2009). Thomas et al. (2018) found that values for HbA1c collected under field conditions in a large population study in Indonesia, with an average temperature above 24°C and humidity of 70%, resulted in upwardly biased estimates of diabetes compared to venous blood or DBS collected under controlled clinic conditions. Vulnerability to degradation of DBS assay values differs by analyte (McDade et al., 2007). For instance, glycosylated hemoglobin (HbA1c) may yield stable values after being stored at room temperature up to a month (Buxton, Malarick, Wang, & Seeman, 2009), whereas C‐reactive protein (CRP) assayed from DBS declines significantly after 2 weeks (McDade et al., 2004).

How multiple preanalytic factors affect the quality and accuracy of DBS samples collected from large population‐based surveys and what analytes are affected by what conditions have not been systematically examined. Most prior studies have attempted to establish the stability criteria focused on relatively small sample sizes, limited to one analyte, or a specific environmental condition, rather than examining how multiple factors associated with collection, storage, transport, and environmental conditions affect the quality of DBS samples used for multiple analytes collected in large population samples. Determining guidelines for preanalytic factors is important in clarifying the validity of DBS‐derived biomarkers and advising studies based on DBS collection.

The present study investigates how preanalytic factors related to collection, storage, transport, and environmental conditions affect the quality and accuracy of DBS samples collected in homes in a nationally representative survey, the Health and Retirement Study (HRS). We take advantage of data from the HRS collected in 2016, when both DBS and VBS were collected on a number of analytes in this large nationally representative population. The availability of analyte results based on VBS allows us to assume that we have a value for the analyte that reflects the actual clinical value; however, we recognize this is a strong assumption given that there is variability over time in the real value of these analytes and that the amount of variability may differ across people. We investigate the links between DBS spot size and quality, shipping time, and atmospheric conditions on the day of collection on the DBS value of analytes while controlling for the value found in venous blood taken an average of 2 months later. We hypothesize that these factors will affect the quality of DBS‐derived biomarkers; and that the effect will be differential by analyte. Our aim is to identify preanalytic factors that affect the quality of DBS samples to promote protocols that produce accurate values of DBS‐derived biomarkers.

## 2 DATA AND METHODS

### 2.1 Data

Data for this study come from the 2016 HRS, a nationally representative survey of the US population over age 50 conducted by the University of Michigan (Juster & Suzman, 1995; Sonnega et al., 2014). HRS collected biomarkers from DBS from a half sample every 4 years from 2006 to 2014 and for the other half sample every 4 years from 2008 to 2016. DBS samples have been assayed for total cholesterol, HDL cholesterol, glycosylated hemoglobin (HbA1c), cystatin C, and CRP since 2006 (Crimmins et al., 2013). In 2016, DBS samples were collected halfway through the household interviews by trained interviewers by filling up to 10 circles with blood droplets across two Whatman blood spot cards. DBS cards were placed in a specially designed cardboard box allowing airflow on all sides for a minimum of 2 hours of drying time prior to shipment. The loaded cardboard boxes were placed in foil pouches with desiccant and then mailed directly to the Department of Laboratory Medicine at the University of Washington in Seattle for assay. On receipt, the lab staff in Washington coded the quality and characteristics of the DBS cards. DBS cards were stored at −70°C prior to and after analysis. The DBS HbA1c assay was performed on a Bio‐Rad Variant II Hemoglobin Testing System (Hercules, CA); assay performance was verified via comparison to HbA1c values of DBS‐matched whole blood samples assayed on that instrument. The DBS total cholesterol assay and the DBS HDL cholesterol assay were developed in‐house using FDA‐cleared reagents from Synermed International (Westfield, IN) and Beckman Coulter (Brea, CA), respectively. CRP was measured in the DBS by an enzyme‐linked immunosorbent assay (ELISA) using FDA‐cleared kits from Biocheck, Inc. (San Francisco, CA). The performance of the DBS assays was verified via comparisons to the concentrations of total cholesterol, HDL cholesterol, and CRP in DBS‐matched plasma samples assayed on a Beckman Coulter AU680 Clinical Chemistry Analyzer. Cystatin C was measured in the DBS by ELISA using INSTAND e.V.‐certified (Düsseldorf, Germany) kits from BioVendor (Brno, Czechia); assay performance was verified via comparison to cystatin C concentrations of DBS‐matched plasma samples assayed on that platform. Commercial certified traceable reference materials were used to monitor the performance of the DBS, blood and plasma assays.

In 2016, HRS collected VBS for the first time. All HRS panel respondents who completed a core interview in the 2016 wave, except proxy respondents and those residing in nursing homes, were asked to consent to a venous blood draw. Venipuncture was performed in a separate home visit by a trained phlebotomist approximately 2 months after the initial visit when the DBS were collected. Fasting was recommended but not required; the fasting status of the donor was noted. The serum separator tubes that contained the samples used for this analysis were centrifuged in the field before being shipped cold to the University of Minnesota Advanced Research and Diagnostic Laboratory where they were processed within 24 hours of arrival or within 48 hours of collection for most respondents. About 92% of the samples arrived at the lab within 24 hours of collection, 99% arrived within 48 hours. Assays were performed on a Cobas 6000 manufactured by Roche. Additional details about the venous blood collection, laboratory equipment, and assay protocol are described elsewhere (Crimmins, Faul, Thyagarajan, & Weir, 2017).

We restricted our sample to those who had both VBS and DBS data where the DBS were collected between April 1, 2016 and December 31, 2016. Because we used the zip code of the respondent's home address to match each person to weather conditions on the day of DBS collection, our analytic sample only included individuals with zip code information. These restrictions left us a sample size of 4456 individuals.

We obtained temperature data from the National Climatic Data Center (NCDC) Global Historical Climate Network Daily (NCDC GHCN‐Daily), which includes daily temperature from approximately 6400 weather stations across the United States. We used the NCDC Local Climatological Data, which contain hourly relative humidity from about 1600 locations across the country, to determine humidity levels. Using ArcGIS we created map layers with point locations representing the geographic locations of weather and humidity stations. Next, we spatially joined weather and humidity data to respondents by determining the nearest station to the respondent using the zip code centroid associated with the respondent's home address where the DBS collection took place. We were unable to obtain temperature or humidity information for 853 individuals because there were no weather recordings available at the nearest station on the date of DBS collection (this was primarily due to DBS collections that occurred on weekends or holidays). Our final analytic sample includes 3321 individuals with slightly different sample sizes for each DBS analyte depending on whether a valid result was obtained.

### 2.2 Measures

#### 2.2.1 Biomarkers

Five biomarkers were assayed at the University of Washington from DBS: total cholesterol, HDL cholesterol, glycosylated hemoglobin (HbA1c), CRP, and cystatin C. These analytes were also collected from VBS. Fasting glucose was measured from VBS as an alternative to HbA1c.

#### 2.2.2 Characteristics of DBS cards and individual blood spots

These were coded by laboratory personnel at the University of Washington. We included the four most frequently used card quality indicators: multiple drops per printed circle, smeared drops/inconsistent absorption, not placing the desiccant correctly for shipping, and overlapping blood spots. Card quality was assessed by the laboratory upon arrival and indicates the quality of the collection by the survey interviewers. Among the spots on cards, the best quality spots were chosen for the assays. Individual spots used for punches for individual assays were also characterized as overlapping/smeared, and small in size. Small spots are blood drops that do not fully fill the preprinted circle on the Whatman 903 collection card used by HRS. A full‐sized drop is ≥1 cm in diameter or about 60 μL of blood; a small spot is <1 cm in diameter, or about ≤40 μL of blood. We also indicated the number of days between collection in the field and receipt at the University of Washington when they were placed in the freezer. We categorized the length of time spent out of the freezer as  0‐2 days, 3 days, 4‐5 days, 6‐7 days, and 8+ days.

#### 2.2.3 Weather conditions

Daytime temperature ≥ 32.2°C (90°F) on the day of DBS collection was categorized as high temperature. We used the highest humidity from noon to 6 pm on the date of collection to indicate the maximum humidity level the DBS was exposed to. A humidity level equal to or greater than 90% was categorized as high humidity.

### 2.3 Statistical model

For each assay, we performed two linear regression models relating the characteristics of DBS cards and drops, to the DBS assay; for the second equation we also controlled for the VBS value. We feel for four of the equations, the VBS value can be regarded as a reliable indicator of the actual value of the assay. Because HbA1c was obtained only from DBS but not VBS, we used the values of glucose measured in serum in the equation estimating HbA1c in DBS. HRS respondents were asked but not required to fast before DBS collection. We restricted our sample to those with a fasted VBS when estimating HbA1c in DBS. All models were estimated in SAS 9.4.

## 3 RESULTS

Descriptive information on potential factors that might affect DBS values is presented in Table 1. Among the indicators of card quality, about 29% had smeared drops and about 20% had multiple drops and overlapping spots. However, because the highest quality spots available were chosen for the assays, overlapping or smeared spots occurred in very few cases for these analytes; only about 3% in most analytes except HbA1c (0.4%). Small spots were much more common; about one‐quarter had small spots for all analytes except for HbA1c (8.2%), which was generally based on the first spot. Maximum temperature on days when DBS were collected ranged from 0°F to 115°F and about 24% were collected on days when the temperature reached 90°F or higher. About 12.7% of the DBS samples were collected on days with maximum humidity greater than or equal to 90%. About 11% spent 0‐2 days in transit from interviewer to the University of Washington; while 20% spent 3 days, 39% spent 4‐5 days and 20% spent 6‐7 days. About 10% did not arrive to the lab for more than a week.

**Table 1:** Percent of samples by characteristics related to validity of DBS assays in the US Health and Retirement Study, 2016 (N = 3321)

	Percent
Quality of DBS cards	
Multiple drops	19.9
Smeared drops	29.0
Overlapping spots	18.5
Incorrectly placed desiccant	5.4
Quality of individual spots	
Overlapping, smeared spot	
Total cholesterol	3.4
HDL cholesterol	3.4
HbA1c	0.4
CRP	2.9
Cystatin C	2.9
Small spot	
Total cholesterol	24.7
HDL cholesterol	24.1
HbA1c	8.2
CRP	26.3
Cystatin C	27.5
High temperature (≥90 °F)	24.2
High humidity (≥90%)	12.7
Days before freezing (DBS)	
0–2 days	10.6
3 days	20.0
4‐5 days	38.6
6‐7 days	20.4
8+ days	10.4

*Abbreviations*: CRP, C‐reactive protein; DBS, dried blood spot; HbA1c, glycosylated hemoglobin; HDL, high‐density lipoprotein.

The mean values, SD, and the range of each biomarker for both DBS and VBS are presented in Table 2. The raw values of DBS‐based total and HDL cholesterol were higher than those from VBS; while the values of CRP and cystatin C were fairly similar to those of VBS, as previously shown in Crimmins et al. (2014). Because of these differences HRS provides both initial DBS values and a value made by equating the distributions to those found in the National Health and Nutrition Examination Survey (NHANES) in the data distributed to users.

**Table 2:** Mean values and range of biomarkers based on DBS and VBS in the US Health and Retirement Study, 2016

	Mean (SD)		Range	
	DBS	VBS	DBS	VBS
Total cholesterol (mg/dL)	274.9 (66.8)	190.2 (42.4)	36‐524	61‐496
HDL cholesterol (mg/dL)	61.8 (20.2)	57.8 (19.6)	10‐189	20‐187
HbA1c (%)	5.6 (0.6)	–	4.1‐17.1	–
Glucose (mg/dL)	–	105.6 (33.6)	–	49‐411
CRP (mg/L)	3.6 (4.6)	3.7 (4.1)	0.1‐20.0	0.2‐20.0
Cystatin C (mg/L)	1.3 (0.5)	1.2 (0.5)	0.1‐7.0	0.5‐9.2

*Abbreviations*: DBS, dried blood spot; HbA1c, glycosylated hemoglobin; HDL, high‐density lipoprotein; VBS, venous blood sample.

Results from regression models are shown in Table 3. Among all analytes, the occurrence of small spots was associated with lower values, though this was only true for HbA1c when the value of glucose was controlled. The reduction was greatest for total cholesterol (42.18 or 63% of a SD); and substantial for HDL cholesterol (6.79 or 34% of a SD), CRP (1.26 or 27% of a SD), and cystatin C (0.20 or 40% of a SD).

**Table 3:** Coefficients from linear regression models estimating value of biomarker from DBS in the US Health and Retirement Study, 2016

	Total cholesterol		HDL cholesterol		CRP		Cystatin C		HbA1c	
	Model 1	Model 2	Model 1	Model 2	Model 1	Model 2	Model 1	Model 2	Model 1	Model 2
Quality of DBS card										
Multiple drops	9.89	7.53	0.04	−2.05	−0.12	−0.15	−0.09	−0.04	0.06	0.15*
Smeared drops	−9.18*	−6.18*	−0.78	0.81	−0.09	−0.09	0.05	0.00	−0.02	−0.03
Overlapping spots	−9.22	−6.15	−0.50	1.59	0.31	0.49	0.09	0.03	−0.08	−0.11
Incorrectly placed desiccant	4.08	−0.89	−2.16	−0.81	0.11	−0.35	−0.01	0.01	0.06	−0.04
Quality of individual spots										
Overlapping, smeared spot	−3.64	−9.77	−1.42	−4.97*	−0.12	−0.41	−0.11	−0.05	−0.12	0.19
Small spot	−42.18*	−39.96*	−6.79*	−10.07*	−1.26*	−0.94*	−0.20*	−0.21*	0.02	−0.09*
Days before freezing (DBS) (ref = 0‐2 days)										
3 days	1.93	2.92	−0.21	−0.69	−0.33	−0.09	−0.01	−0.00	0.09*	0.08*
4–5 days	−5.90	−4.75	−2.48*	−1.75	0.08	0.14	−0.00	−0.03	0.06	0.08*
6–7 days	−11.19*	−12.97*	−2.70*	−1.88	−0.48	−0.29	0.05	−0.03	−0.04	0.00
8+ days	−41.46*	−42.99*	−10.62*	−10.09*	−0.55	−0.51	−0.07	−0.11*	0.01	−0.01
High humidity	−8.70*	−11.80*	−2.10	−4.56*	0.35	0.28	−0.03	−0.02	−0.06	−0.05
High temperature	−13.22*	−7.64*	−5.91*	−5.10*	0.07	−0.27	0.07*	0.01	0.05	−0.04
VBS		0.80*		0.59*		0.63*		0.84*		0.01*
Intercept	301.07*	146.24*	68.44*	34.91*	4.10*	1.70*	1.35*	0.43*	5.57*	4.21*
N	3110	3109	3060	3060	3158	3156	3149	3149	2857	1874
*R* ^2^	0.13	0.39	0.06	0.36	0.02	0.35	0.04	0.78	0.01	0.41

* *P* < .05.

*Note*: Model 1, effect on DBS value; Model 2, effect on DBS value adjusted by VBS.

*Abbreviations*: CRP, C‐reactive protein; DBS, dried blood spot; HbA1c, glycosylated hemoglobin; HDL, high‐density lipoprotein; VBS, venous blood sample.

None of the other quality factors was significantly associated with the CRP DBS value except the VBS value. The HbA1c value was also not significantly affected by these factors: multiple drops are associated with higher values whereas longer shipping time, 3‐5 days, appears to increase the value. Cystatin C values also appear little affected by additional factors: there is a small but significant increase in value on hot days and a decrease for a shipping time of 8+ days. For these three analytes, only a small portion of the variance is explained by these conditions: 2% for CRP, 4% for cystatin C, and 1% for HbA1c.

Total and HDL cholesterol values based on DBS were fairly strongly affected by the number days they were in transit before arrival at the lab and weather conditions (Table 3). Shipping of 6 to 7 days resulted in a drop of 11.19 mg/dL in the value of total cholesterol and 2.70 mg/dL in HDL cholesterol. About 10% of the sample experienced shipping time of 8 days or more and this was linked to total cholesterol 41 mg/dL lower and HDL cholesterol 11 mg/dL lower than if shipping had been accomplished in 2 or fewer days. Both high temperature and high humidity were linked to lower values of DBS total and HDL cholesterol.

## 4 DISCUSSION

This study examined how factors related to DBS collection, shipping, and environment conditions influence the validity of DBS assays in a large, geographically diverse population sample. While DBS is a popular method for collecting biomarker data in large surveys, our general conclusion is that this method of collecting blood can be potentially vulnerable to collection method and conditions. We can draw several specific conclusions from our work. First, the effect of collection, shipping, and environmental factors on DBS samples varies by analyte. Stability needs to be evaluated for each analyte of interest as the biochemistry of different analytes is likely to produce a variety of patterns of stability. Cholesterol measures in particular are sensitive to multiple influences including quality of DBS cards, spots, shipping time, and weather. Adverse circumstances generally reduce the values of the analytes. Cholesterol assays will have questionable validity where spots are small, shipping time is long and they are exposed to heat and humidity. On the other hand, CRP, cystatin C, and HbA1c were relatively insensitive to shipping and weather.

Second, small spots are a problem which should be avoided as they lower the values of all DBS measures examined here. Instructions to avoid small spots need to be reinforced with sample collectors. In addition, sample spots should be coded upon receipt so an adjustment for small spots can be made. The overall quality of the cards and the use of a smeared spot seemed to have little effect on the resulting DBS values. Our third conclusion is that weather conditions do not generally have significant effects on these assays; with the exception of cholesterol.

The results of this study suggested the importance of documenting information on the quality of DBS spots as well as shipping time and conditions. While it is important to emphasize a protocol to minimize the effect of these factors, there are circumstances when conditions are not controllable, and having information on these factors can be used for adjustments to assay values or included in analytic models. We should note that the inclusion of what we considered the gold standard, VBS value, did not change our results significantly meaning that adjustments could be made without this value. We also reiterate that the fact that there was a two‐month average time difference between the collection of the DBS and the collection of blood for VBS was not ideal.

This analysis is based on blood spots that were being collected for the sixth time in the HRS. Other studies may have similar, or even worse, problems with card and spot qualities. Continued use of the valuable DBS technology requires more validity and quality testing for other assays.

## CONFLICT OF INTEREST

The authors declare no potential conflict of interest.

## AUTHOR CONTRIBUTIONS

E.C. designed the study and directed the analyses; J.K.K. ran all analyses; Y.Z., J.K.K., S.F., H.K. and H.S. prepared the data. J.A., A.P., J.C., and J.F. contributed to the acquisition of data. All authors edited, critically reviewed, and approved the final content of the manuscript.
